# Models and tissue mimics for brain shift simulations

**DOI:** 10.1007/s10237-017-0958-7

**Published:** 2017-09-06

**Authors:** Antonio E. Forte, Stefano Galvan, Daniele Dini

**Affiliations:** Department of Mechanical Engineering, Imperial College London, London, SW7 2AZ UK

**Keywords:** Brain phantom, FE modelling, Soft tissue, Biomechanics, Brain tissue, Image-guided surgery

## Abstract

Capturing the deformation of human brain during neurosurgical operations is an extremely important task to improve the accuracy or surgical procedure and minimize permanent damage in patients. This study focuses on the development of an accurate numerical model for the prediction of brain shift during surgical procedures and employs a tissue mimic recently developed to capture the complexity of the human tissue. The phantom, made of a composite hydrogel, was designed to reproduce the dynamic mechanical behaviour of the brain tissue in a range of strain rates suitable for surgical procedures. The use of a well-controlled, accessible and MRI compatible alternative to real brain tissue allows us to rule out spurious effects due to patient geometry and tissue properties variability, CSF amount uncertainties, and head orientation. The performance of different constitutive descriptions is evaluated using a brain–skull mimic, which enables 3D deformation measurements by means of MRI scans. Our combined experimental and numerical investigation demonstrates the importance of using accurate constitutive laws when approaching the modelling of this complex organic tissue and supports the proposal of a hybrid poro-hyper-viscoelastic material formulation for the simulation of brain shift.

## Introduction

The human brain undergoes deformation when a craniotomy of considerable size is performed during surgery. The phenomenon is usually identified as brain shift and is due to a variety of reasons including gravity, pharmacologic responses, surgical manipulation (Nabavi et al 2001; Nimsky et al. 2001; Roberts et al. 1998). In particular, the loss of cerebrospinal fluid (CSF) during surgery, and consequentially of buoyancy forces surrounding the brain, is recognized as the main cause of brain shift (Dumpuri et al. 2007; Roberts et al. 1998). It has been shown that brain can shift up to twenty millimetres in a non-rigid fashion (Hartkens et al. 2003). This introduces a non-negligible error in targets location, which results in lowering the accuracy of surgical procedures. Surgeons try to compensate for brain shift with their own experience, relating locations to anatomical features in order to follow targets inside the brain. In extreme cases, intraoperative magnetic resonance images (MRIs) are used to relocate targets and compensate for excessive deformations (Nimsky et al. 2000). Unfortunately, portable MRI scanners are expensive, have restricted surgical access and are currently not available in the majority of the facilities (Škrinjar et al. 1998). In addition, intraoperative scans tend to prolong the surgery, introducing additional risks for the patient. Therefore, there is a need for tools able to accurately predict brain shift pre-operatively and/or offer real-time guidance to the surgeon during the procedures.

Real-time algorithms running on both CPUs and GPUs have been extensively discussed in literature showing promising results (Archip et al. 2007; Dumpuri et al. 2007; Joldes et al. 2009a, c; Škrinjar et al. 2002, 1998; Warfield et al. 2002). In most cases, finite element models are used to compute 3D deformation fields (of the whole brain) resulting in the imposition of intraoperative shift measurements (provided as input) of the exposed brain cerebral cortex. Both linear (Archip et al. 2007; Škrinjar et al. 2002; Warfield et al. 2002) and nonlinear finite element algorithms (Joldes et al. 2009a, c) have been implemented for these purposes. The resultant displacement field is then used to deform the pre-operative MRIs offering guidance to the surgeon in real time. Although these tools provide guidance capabilities in the range of the neurosurgery requirements, compromises are needed for meeting real-time performances: coarse mesh resolution (Hu et al. 2007; Joldes et al. 2009a; Škrinjar et al. 2002; Wittek et al. 2007), simplified boundary conditions (for example constrained degrees of freedom for simulating the falx cerebri and braincase) (Dumpuri et al. 2007; Joldes et al. 2009a; Škrinjar et al. 2002), and /or simplified material formulations (Ferrant et al. 2001, 2002; Škrinjar et al. 2002, 1998; Warfield et al. 2002). Furthermore, intraoperative sensors such as stereo-cameras, ultrasound scans (US) or laser range scanners (LRS) are needed in order to provide the correct input (i.e. measured shift at the craniotomy site) to drive the model (Rasin et al. 2014).

Fewer examples of not displacement-driven approaches are provided in the literature (Dumpuri et al. 2003; Hu et al. 2007). These models are usually identified as gravity-driven models since the deformation is induced by a gravity load. Because of the lack of intraoperative measurements providing inputs to the model, accurate boundary conditions, geometries and material properties are needed for reproducing the complex phenomena realistically.

Wittek et al. (2009) showed that material properties and formulations are of little importance when using monophasic, incompressible, displacement-driven models. However, the same conclusion is (i) not immediately evincible for biphasic models, where results are affected by the compressibility of both the phases (Forte et al. 2015) and (ii) not applicable to load-controlled models (i.e. gravity-driven models). Ruling out basic material descriptions [monophasic linear elastic (Ferrant et al. 2001, 2002; Warfield et al. 2002) and linear viscoelastic (Škrinjar et al. 1998, 2002) formulations], which are ineffective in gravity-driven models, there is still an open debate on the what is the best material formulation for modelling brain shift, with the use of biphasic poroelastic or monophasic nonlinear viscoelastic constitutive laws being favoured for reproducing the brain tissue mechanical behaviour.

Dumpuri et al. (2003, 2007) proposed a linear poroelastic formulation based on the biphasic consolidation theory. The atlas of boundary conditions presented in their works involves different patient orientations and CSF amounts in order to compute pre-operatively a number of possible scenarios. However, geometrical details are not reproduced (e.g. sulci and gyri on the cerebral cortex) and the model is spatially constrained to simulate the presence of the braincase instead of using more realistic contact algorithms for the brain–skull interface.

Miller (1998) asserted that a biphasic poroelastic model is not suitable for mimicking brain tissue. This is based on the observation that such models cannot simulate the large relaxation ratios observed in the brain tissue (Cheng and Bilston 2007; Forte et al. 2016b). Therefore, Miller and collaborators developed a nonlinear viscoelastic material model based on experimental tests on swine brain tissue, (Miller and Chinzei 2002). However, this monophasic approach lacks of solid–liquid interaction properties that might be relevant when simulating gravity-driven brain shift phenomenon. In fact, Bilston et al. (1997) found that brain tissue lacks a long-term elastic modulus and thus can be treated as a fluid. In Cheng and Bilston (2007) carried out an experimental campaign on calf brains, focusing in particular on the white matter, and showed that the poro-viscoelastic model provided the best match for brain tissue. Similar results have been obtained for liver tissue (Raghunathan et al. 2010). Additionally, Franceschini et al. (2006) demonstrated that brain tissue obeys to the biphasic consolidation theory and that viscous components are present in the solid phase. Although these works have proved the importance of considering both the solid matrix viscoelasticity and the solid–fluid interactions when modelling brain tissue, there is no study that clarifies the importance of an accurate material formulation in gravity-driven brain shift simulations.

Here, considering the most promising of the constitutive material descriptions proposed by the other research groups previously mentioned, we implement and compare a hyper-viscoelastic (HVE), a poro-hyperelastic (PHE) and a hybrid poro-hyper-viscoelastic (PHVE) formulation, with the aim to clarify the role of constitutive material laws in the prediction of the brain shift phenomenon. Thanks to our previous efforts in designing a new composite hydrogel (CH), which is suitable for reproducing complex deformation scenarios (e.g. brain shift) and mimicking the organic tissue’s mechanical behaviour in compression, indentation, relaxation, hysteresis and shear (the hydrogel is in fact much softer than the silicon Sylgard gel used, for example, by Destrade et al. (2015)), and a life-sized phantom that can reproduce brain shift monitoring the level of CSF left in the skull (Forte et al. 2016a), we are now able to evaluate our models by the means of a controlled experimental apparatus. This enables the comparison of constitutive material descriptions, ruling out the spurious effects related to patient variability in terms of geometric features and tissue properties, CSF amount uncertainties and head orientation. Although Feng et al. (2017) showed that brain tissue white matter exhibits transverse isotropy characteristics, the hydrogel used hereby is isotropic.

**Fig. 1:**
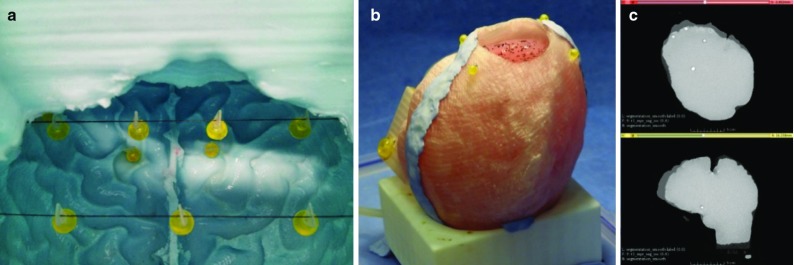
**a** High visibility markers for MRI positioned inside the mould of the phantom before pouring the composite hydrogel for casting; **b** the physical model with the mock-up skull sealed and filled with water; **c** the same phantom segmented in the MR images. The PinPoint 187 markers are clearly visible (*white dots*)

The direct comparison between the MRI-based deformation data in the synthetic phantom and numerical models obtained from the same pre-operative information used to produce the brain–skull mimic allow for the first time a direct assessment of the accuracy that different constitutive laws can achieve in reproducing 3D deformation patterns. Differences in the material response linked to different formulations are highlighted, proving that a hybrid biphasic, nonlinear viscoelastic model is able to accurately reproduce the complex mechanical behaviour of the hydrogel, which in turn has been shown to replicate brain deformation within a certain range of loading conditions. Given the analogy between the mechanical behaviour of the synthetic tissue and real brain, our results support previous findings by Bilston et al. (1997), Cheng and Bilston (2007) and Franceschini et al. (2006), whose work encourages the use of biphasic nonlinear formulations for the accurate prediction of brain tissue response.

## Materials and methods

### Phantom

A novel composite hydrogel as a substitute of the real brain tissue for testing and validation purposes has been previously designed (Forte et al. 2016a). The hydrogel is capable of reproducing the rate-dependent mechanical response of brain tissue in compression, indentation, relaxation, hysteresis and shear. The hydrogel can be cast in the shape and size of a human brain and used together with a plastic mock-up skull (Cattilino et al 2014), composing a brain–skull phantom (Fig. 1b). Details of the making procedure are reported in (Forte et al. 2016a).

The validation/characterization of the model was performed comparing the deformation measured with the phantom under brain shift conditions (at different levels of CSF) and the deformation predicted by the model. The three-dimensional deformation field reproduced by the phantom was measured via MRI scans.

The composite hydrogel used for the brain phantom is slightly denser than water (density average value and standard deviation: $1015\,\pm \, 13\,\hbox {Kg}\,\hbox {m}^{-3}$, calculated on 5 samples). The small difference in density between the two mediums (i) avoids flotation and (ii) do not induce significant preloading to the phantom brain when completely submerged.

**Fig. 2:**
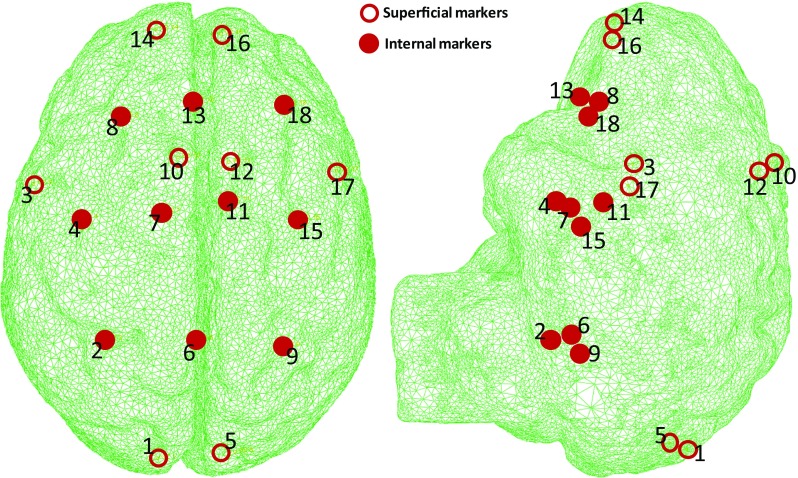
PinPoint 187 MRI markers positions inside the brain phantom meshed geometry shown after reconstruction via MRI segmentation

The hydrogel produces a homogeneous phantom. As a consequence, it is not possible to identify any intrinsic feature inside its volume to track among several MRI acquisitions. For this reason, MRI compatible markers PinPoint 187 (Beekley Corp, Bristol, CT, USA) were embedded in the phantom. These markers are designed for diagnostic purposes and are highly visible in the images, allowing easy identification (Fig. 1c). Their weight is negligible if compared to the brain phantom. The markers have similar density ($1041\,\pm \,2\,\hbox {Kg}\,\hbox {m}^{-3}$, average and standard deviation) to the phantom material ($1015\,\pm \, 13\,\hbox {Kg}\,\hbox {m}^{-3}$), which is close to that of water ($1000\,\hbox {Kg}\,\hbox {m}^{-3}$). We can therefore assume that the material distortion caused by the markers is very limited. A number of markers can be placed inside the phantom without altering its mechanical response and weaken the overall structure. The positioning of the markers was carefully chosen in order to obtain shift measurements in several locations. In the final set-up, 18 markers were arranged inside the mould. Eight were placed close to the surface, in symmetric positions with respect to the falx cerebri: two in the anterior area of the frontal lobe, two in the superior area of the frontal lobe, two in the temporal lobe and two in the occipital lobe (superficial markers in Fig. 2). The remaining markers were placed in the middle of the volume by hanging them to thin cotton threads that were pulled taut and fixed to the junction of the two halves of the mould (Fig. 1a). The arrangement consisted of three rows composed by three, four and three markers, respectively (internal markers in Fig. 2). The phantom preparation started 2 days before the acquisition. Before the MRI scanning, the phantom was carefully placed inside the mock-up skull, assuring the correct positioning of the tissue inside the plastic skull-shaped container. Afterwards, the skull was sealed watertight using putty. The physical model was then filled with water, to simulate the presence of the CSF. The skull presents a craniotomy performed according to the specifics of the surgeon, not relevant for this work. The apparatus was completely submerged in a water bath, with both the craniotomy (placed on the top of the skull) and the hole for the water drainage (placed on the bottom of the skull) open. This forced all the air to leave the set up and get replaced by water.

**Fig. 3:**
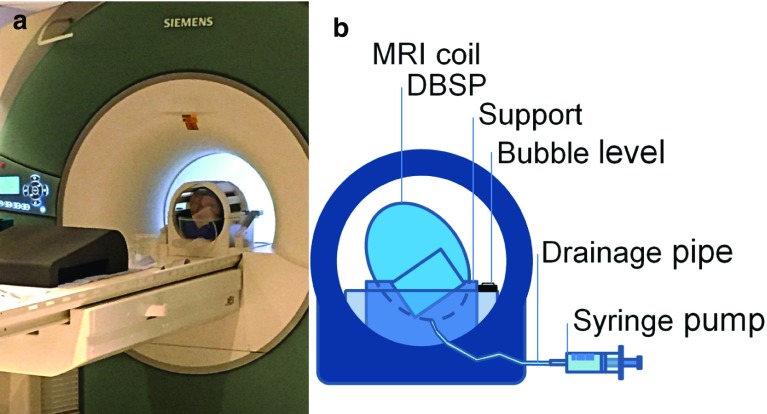
**a** The complete phantom set-up positioned on the MRI table before the acquisition begins; **b** schematic of the set-up showing the details of the test configuration

The craniotomy was closed and sealed in order to achieve a 100% level of fluid inside the skull. The physical model was fixed into a shallow transparent box to prevent leakage of water inside the MRI scanner. A small plastic bubble level was used to check the levelling of the set-up. The complete set-up was then transported to the Department of Psychology at the Royal Holloway University of London. The phantom was placed inside a 32-channel array head coil and placed on the examination table inside the 3-Tesla Magnetom Trio scanner (Siemens AG, Munich, Germany) as depicted in Fig. 3a and b. The type and size of the coil was chosen in order to be able to contain the physical model while maintaining a good signal-to- noise ratio.

#### Data acquisition

Structural data were acquired using a T1-weighted 3D anatomical scan (MPRAGE, Siemens, TR 1830 ms, TE 5.56 ms, flip angle $11{^{\circ }}$, scan resolution 0.75 $\times$ 0.75 $\times$ 1.0 mm). In previous tests, this modality showed the best results in terms of resolution and contrast between the phantom and the liquid.

The phantom was equipped with a long draining pipe to allow the manual draining while keeping the MRI table in position for the scanning. This prevented motion of the set-up and oscillation of the liquid inside the skull. Doing so, all the images are inherently aligned with no need for rigid transformations during post-processing. This solution prevents the introduction of additional inaccuracies due to data manipulation. The amount of water drawn was controlled using a syringe with a volumetric scale.

The initial acquisition was performed with the skull completely full of liquid (100%, 220 ml of water). For the second acquisition, the volume of the fluid was decreased by 40 ml. The amount liquid left in the skull after the first drainage (180 ml) represents the maximum level of water when the craniotomy is exposed. The subsequent six scans were taken at constant draining steps of 30 ml (water left in the skull: 150, 120, 90, 60, 30, 0 ml). During the draining steps, the craniotomy was left open in order to allow air inside the skull, which would replace the drawn water content.

Despite common practice for medical examinations, the image volume was not aligned to the anatomical orientation of the head. Thus, the images represent the actual orientation of the set-up, and hence of the phantom, inside the MRI scanner. This is useful when setting up the FE model in order to easily identify the direction of the gravity vector with respect to the phantom. With this approach, the gravity vector simply points downwards.

#### Data analysis

The data collected were analysed to extract information for the characterization and validation of the finite element model. The first acquisition (at 100% fluid level) was processed using 3D Slicer (Fedorov et al. 2012), and the volumetric shape of the phantom was segmented from the complete volume. To this end, an extension of 3D Slicer called CarreraSlice (Carrera Slice module for assisted segmentation in the 3D Slicer software suite) was used. CarreraSlice is an interactive 3D segmentation tool that performs semi-automatic segmentations on the basis of human input and refinement. The segmented model was first resampled to an isotropic voxel size of 1.0 $\times$ 1.0 $\times$ 1.0 mm and subsequently used to generate a tetrahedral mesh as input geometry in Abaqus (Dassault Systemes Simulia Corp, Providence, RI, USA). The brain phantom model was imported keeping its orientation consistent with the data acquired in the MRI and to permit to define the gravity vector correctly.

**Fig. 4:**
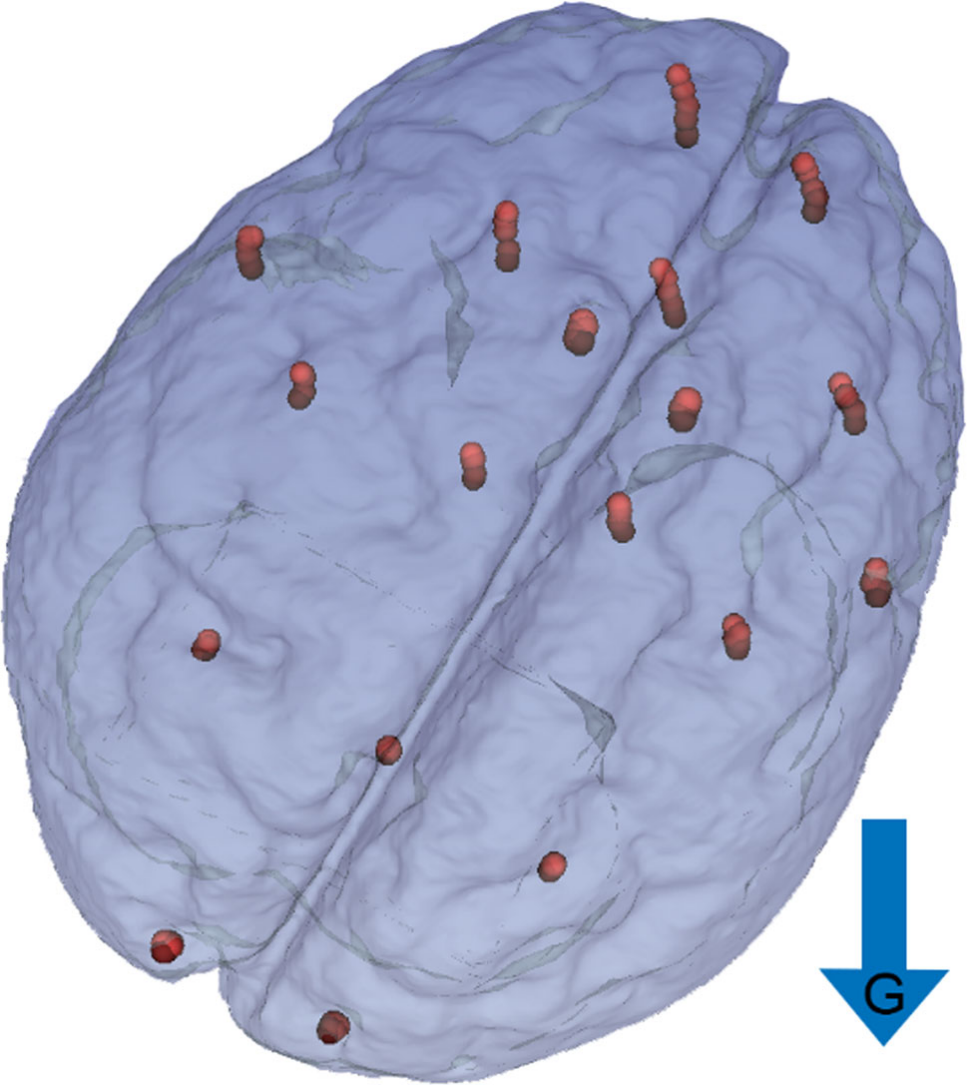
Evolution of the MRI markers positions during five acquisition steps; the *arrow* represents the direction of the gravity vector

The positions of the centroids (mean millimetric coordinates of constituent voxels) of the PinPoint 187 markers were extracted from the images. Figure 4 shows the position of the markers for five consecutive acquisitions. It is worth noticing how different areas of the brain phantom deform in different ways under gravity.

### Model

The model of the apparatus designed in Abaqus included both brain and skull geometries (Fig. 6a). The brain mesh had 94,211 porous tetrahedral elements (C3D4P for PHVE and PHE, C3D4H for the HVE formulations) and about 36 nodes per $\hbox {cm}^{2}$ on the surface, assuring a detailed reproduction of the phantom cerebral cortex geometric features (sulci and gyri). The skull had 117,799 rigid triangular shell elements (R3D3). An “encastre” boundary condition was applied to the skull reference point, fixing all the possible degrees of freedom. Mesh convergence tests were performed to validate the final mesh density which was chosen when results deviated by only 1% in terms of maximum deformation recorded for all markers. The phantom brain was able to shift inside the skull and to detect the physical boundary by means of a contact interaction algorithm. Furthermore, tangential friction effects were added between brain and skull and the brain and the falx geometry to enhance the reality in the deformation patterns (Fig. 6a). Frictional experiments were run to measure the friction coefficient at the hydrogel/skull interface (friction coefficient = 0.5; results not reported for brevity).

#### Material characterization for constitutive laws

Compression–relaxation tests were carried out on hydrogel cylindrical samples casted from the same polymeric solution we used for the life-sized phantom. The Mach-1$^{\mathrm{TM}}$ mechanical testing system (Biomomentum, Canada) was chosen as testing rig for the compression tests. A 1.5 N single-axis load cell was used to measure the vertical force ($75\,\upmu \hbox {N}$ resolution), and the vertical displacement was measured by the moving stage of the rig ($0.1\,\upmu \hbox {m}$ resolution). Silicon oil with a kinematic viscosity of $5\times 10^{-4}\,\hbox {m}^{2}/\hbox {s}$ was applied at the interface between the sample and the compression platens in order to minimize friction effects (Charalambides et al. 2001, 2005; Leibinger et al. 2015). Eight cylindrical samples were tested ($12\,\pm \,1\,\hbox {mm}$ diameter, $7\,\pm \,1\,\hbox {mm}$ height), and the results were averaged. No pre-conditioning was performed, and only one loading cycle was executed on each sample. The specimen was left for 1 min before the actual test began to obtain stable measurements of load and height and then compressed at constant velocity until a displacement corresponding to 30% of the measured height (equal to 0.356 true strain) was achieved. Afterwards, a relaxation step of 500 s was applied by holding the upper plate at the maximum strain value. The tests were conducted at the “instantaneous” velocity of 8.3 mm/s. All the tests were performed in a conditioned room at $19 {^{\circ }}\hbox {C}$ temperature.

The hydrogel was modelled as a hyper-viscoelastic “sponge-like” porous matrix saturated with water (PHVE). In fully saturated conditions, the total stress in the medium at a point, $\sigma$, is given by:

$$\begin{aligned} {\varvec{\sigma }} ={\bar{{\varvec{\sigma }}}}^{*}-p{{\varvec{I}}} \end{aligned}$$

where ${\bar{\sigma }} ^{*}$ is the effective stress in the porous material skeleton, *p* is the pressure stress in the wetting liquid and ${{\varvec{I}}}$ is the identity matrix. The rate-independent response of the solid skeleton was assumed to follow the Ogden hyperelastic model. This model has a strain energy potential *U* defined as:

1 $$\begin{aligned} U=\frac{2\mu _0 }{\alpha ^{2}}\left( {\bar{\lambda }} _1^\alpha +\,{\bar{\lambda }}_2^\alpha +{\bar{\lambda }}_3^\alpha -3 \right) +\frac{1}{D}\left( {J-1} \right) ^{2} \end{aligned}$$

where ${\bar{\lambda }}_i$ are the deviatoric principal stretches and they are equal to ${\bar{\lambda }}_i =J^{-\frac{1}{3}}\,\lambda _i$; $\lambda _i$ are the principal stretches;$\,\mu _0 , \alpha$ and *D* are material parameters; and *J* is the volume strain (equal to $\lambda _1 \lambda _2 \lambda _3 )$. The stresses are then given by partial differentiation of Eq. (1), i.e. ${\bar{{\varvec{\sigma }}}} ^{*}=\frac{\mathrm{d}U}{\mathrm{d}\lambda }$.

Note that $\mu _0$ is the instantaneous shear modulus, whereas the bulk modulus *K* is related to *D* and Poisson’s ratio, $\nu$, through:

2 $$\begin{aligned} D=\frac{2}{K}=\frac{3\left( {1-2\nu } \right) }{\mu _0 \left( {1+\nu } \right) } \end{aligned}$$

The rate-dependent response of the solid matrix is implemented in the model defining the shear stress ($\tau \left( t \right)$) relation for a viscoelastic model:

$$\begin{aligned} \tau \left( t \right) =\int _0^t \mu \left( {t-s} \right) {\dot{\gamma }}\left( s \right) \mathrm{d}s \end{aligned}$$

where ${\dot{\gamma }}$ is the shear strain rate and $\mu \left( t \right)$ is the time-dependent shear relaxation modulus which can also be written as:

3 $$\begin{aligned} \mu \left( t \right) =\mu _0 g_R \left( t \right) \end{aligned}$$

where $\mu _0$ is the instantaneous shear modulus mentioned above, which represents the shear relaxation modulus when $t=0$. Using a Prony series, one can obtain:

$$\begin{aligned} g_R \left( t \right) =1-\sum _{i=1}^N g_i \left( {1-e^{-\frac{t}{\tau _i }}} \right) \end{aligned}$$

$g_i ,$ and $\tau _i$, are the Prony constants and the retardation time constants, respectively. Therefore, Eq. (3) becomes:

$$\begin{aligned} \mu \left( t \right) =\mu _0 \left( {1-\mathop \sum \limits _{i=1}^N g_i \left( {1-e^{-\frac{t}{\tau _i }}} \right) } \right) \end{aligned}$$

A liquid phase (incompressible by default, $\nu = 0.5$) is also present in the formulation. The fluid flow is governed by Darcy’s law:

4 $$\begin{aligned} n{{\varvec{v}}}=-\frac{k}{\gamma _w }\left( {\nabla p-\rho _w {{\varvec{g}}}} \right) \end{aligned}$$

where ${\varvec{v}}$ is the fluid flow velocity vector, *n* is the porosity of the medium, $\gamma _w$ is the specific weight of the fluid, $\nabla p$ is the pressure gradient vector, *k* is the hydraulic conductivity of the medium,$\,\rho _w$ is the fluid density and ${{\varvec{g}}}$ is defined as the gravitational acceleration vector:

$$\begin{aligned} {{\varvec{g}}}=-g\nabla z \end{aligned}$$

where *g* is the gravitational constant (=$9.812\,\hbox {m/s}^{2}$) and *z* is the elevation above some datum. Note that the hydraulic conductivity, *k* (units m/s), is related to conventional permeability, $\Pi$ (units $\hbox {m/s}^{2})$, through:

$$\begin{aligned} \Pi =k\frac{\eta }{\rho _w g} \end{aligned}$$

As already mentioned, the hydrogel is assumed to be fully saturated with water, i.e. all voids in the material are filled up with the wetting liquid. In addition, the void ratio *e* is defined as the ratio of volume of wetting liquid $$V_w $$ to the sum of the volumes of the solid $$V_s $$ and trapped liquid $$V_t $$:

$$\begin{aligned} e=\frac{V_w }{V_s +V_t } \end{aligned}$$

Therefore, the porosity *n*, in Eq. (4) is related to void ratio, *e*, through:

$$\begin{aligned} n=\frac{e}{1+e} \end{aligned}$$

**Fig. 5:**
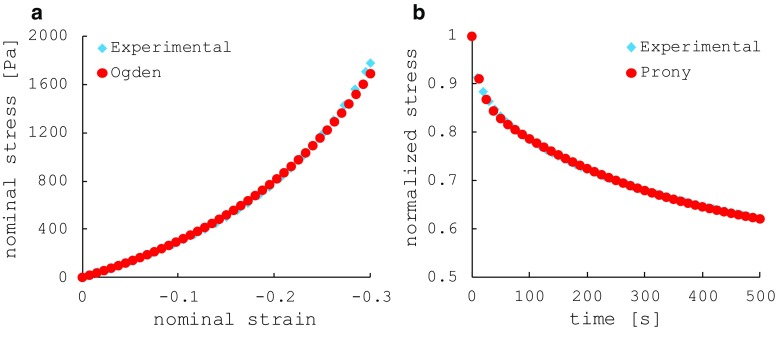
Fitting analysis on the experimental compression (**a**) and relaxation (**b**) test carried out on hydrogel cylindrical samples

#### Model steps definition

**Fig. 6:**
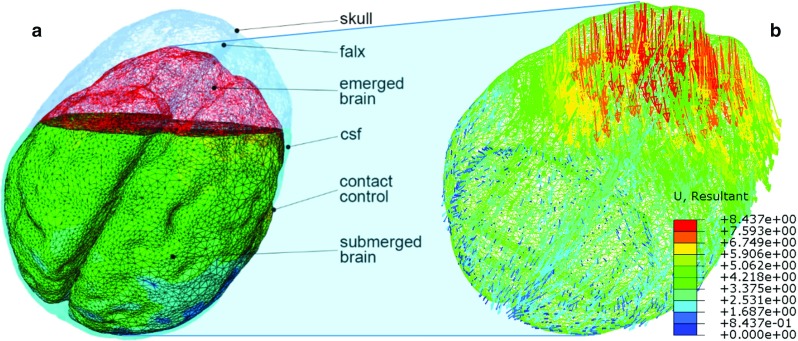
**a** High resolution model. Particular of emerged and submerged areas of the brain phantom, “CSF” level, skull and falx rigid geometries interacting with the brain via normal and tangential contact controls; **b** vector plot of the resultant displacements in mm (PHVE model)

The model was built to simulate five subsequent draining configurations corresponding to the water levels taken from the MRI scans. In particular, starting from the fully filled initial configuration (100% of water in the skull corresponding to 220 ml and 0% of CSF loss) the model can sequentially reproduce 18, 32, 46 and 60% of liquid loss which are, respectively, 180, 150, 120 and 90 ml of liquid left in the skull. The CSF volume left in the skull after each draining step during the scans was calculated by subtracting the amount of water drained out of the skull (measured with a scaled syringe attached to the drainage pipe) from the total CSF volume. The level of the liquid left inside the skull was also double-checked with the MRI scans at each draining step (the liquid is visible in the images). Based on consultations with a specialized surgeon, we considered a loss of 60% of CSF an extreme condition during real surgeries; therefore, five steps are sufficient for the simulation to cover any possible scenarios. The model allows association of every step with the corresponding changes in boundary conditions. In particular, the model takes into account loss of buoyancy forces, occurring of the gravity load and free draining conditions for the regions of the brain above the water level (emerged regions, see Fig. 6a). Each step had three sub-steps in order to (i) facilitate the convergence of the solver and (ii) reproduce the CSF draining procedure as it had been performed during the acquisitions. The first sub-step consisted of an initial soils step where the gravity load takes place and an initial stabilization of the pore pressure distribution occurs in the phantom (duration = 0.1 s). In the second sub-step (duration = 10 s), the pore pressure boundaries are released (imposing the pore pressure = 0) at the emerged nodes of the phantom brain, activating the free drainage condition that allows the liquid phase to flow, move inside and gradually leave the phantom. This approximately corresponds to the actual time we took for draining the water out of the skull using the syringe pump. The free draining boundary gives an additional capability to both the poro-hyper-viscoelastic and the poro-hyperelastic formulations since the overall Poisson’s ratio of the emerged areas of the brain will vary over time (according to the permeability *k*) as the fluid phase leaves the interested areas. The variability range of $\nu$ spans from 0.5 to 0.35 that represent the fully saturated condition (fluid compressibility leads) and the drained condition (fluid has left, solid matrix compressibility leads), respectively.

**Table 1:** Summary of the material coefficients in Abaqus format for implementing the three different material formulations

	Solid phase							Fluid phase		
	Rate-dependent				Rate-independent					
Model	$g_{1}$	$g_{2}$	$\tau _{1} (\hbox {s})$	$\tau _{2} (\hbox {s})$	$\mu _{0} (\hbox {Pa})$	$\alpha$	$D\,(\hbox {Pa}^{-1})$	$k\,(\hbox {m s}^{-1})$	$e_{0}$	$\gamma _{w } (\hbox {N m}^{-3})$
PHVE	0.13	0.32	14	333	794.36	$-$2.8	0.84E$-$03	1.57E$-$9	0.2	9779
PHE	$-$	$-$	$-$	$-$	794.36	$-$2.8	0.84E$-$03	1.57E$-$9	0.2	9779
HVE	0.13	0.32	14	333	794.36	$-$2.8	0	$-$	$-$	$-$

An additional sub-step of 60 s was added to take into account the time for leaving the MRI room and starting of the scanning procedure. The nearest nodes of the mesh to the PinPoint 187 markers were selected minimizing the distance vector between the node coordinates and the marker centroid coordinates.

## Results

### Material characterization

The average compression curve obtained from the experimental tests was fitted with the Ogden hyperelastic formulation (Fig. 5a) (Miller and Chinzei 2002). The material parameter $\alpha$ and the initial (or instantaneous) shear modulus $\mu _0$ in Table 1 were found at this stage. Furthermore, a Prony series was fitted on the relaxation part of the unconfined compression curve (Fig. 5b). The Prony series method is widely used in FE analysis software in order to reproduce the dynamic behaviour of materials by scaling the stiffness according to the strain rate applied. A minimum of two pairs of material parameters ($g_{k}$ and $\tau _{k}$) were needed for replicating the rate-dependent response of the hydrogel. Assuming that the hydrogel has similar compressibility, permeability and void ratio of brain tissue, these additional parameters were obtained from the literature. As suggested by Kaczmarek et al. (1997), $\nu = 0.35$ was assumed as Poisson’s ratio of the solid matrix and *D* was calculated according to Eq. (2). This value represents only the relative compressibility of the material’s solid phase, which allows fluid to be absorbed or exuded from the solid matrix (Taylor and Miller 2004). Using the permeability option in Abaqus, a liquid phase (incompressible by default, $\nu = 0.5$) was introduced in the formulation. The definition of a biphasic model enables a second rate dependency caused by the movement of the fluid within the solid matrix. The difference in compressibility of the two phases partially monitors this rate dependency, as shown in (Forte et al. 2015). To complete the definition of the fluid phase, the liquid was treated as water, defining the specific weight ($\gamma _{w}$) accordingly. The permeability value (*k*) was obtained from (Kaczmarek et al. 1997) and the initial void ratio of the material ($e_{0}$) from (Nagashima et al. 1987). The material coefficients are summarized in Table 1.

The presented poro-hyper-viscoelastic formulation can be easily implemented in Abaqus and simulated by using the “soils transient consolidation” step. The poro-hyperelastic formulation was obtained neglecting the viscoelasticity of the solid matrix by removing the Prony series from the material definition. For the hyper-viscoelastic formulation, the fluid phase was omitted removing permeability and void ratio from the material definition and imposing $\nu = 0.5$ to the solid phase. The Abaqus “visco” step was used in this last case.

### Brain shift simulations

**Fig. 7:**
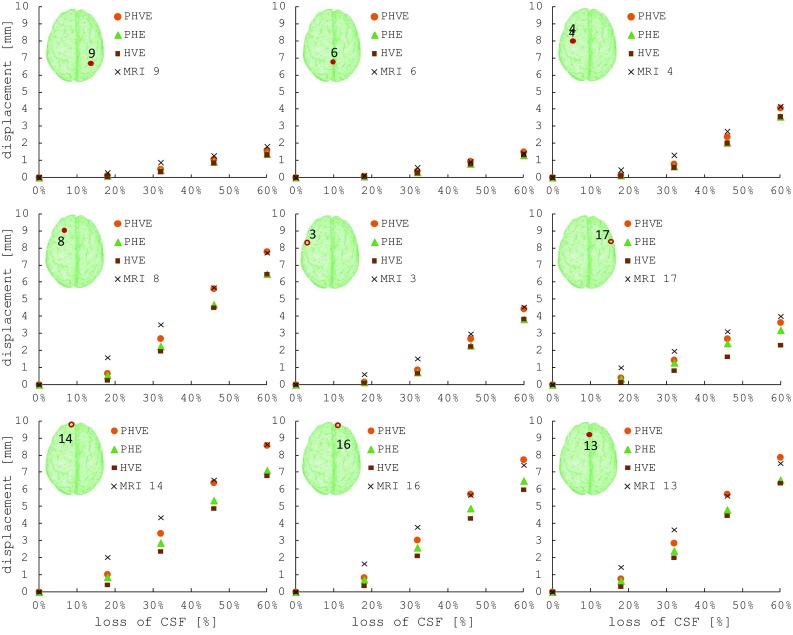
Comparison between magnitude of displacement measured in the MRI scans and the model results (three material formulations) for each marker at each draining step (percentage of the volume of CSF lost at each step)

The deformation computed by the model (example reported in Fig. 6b) was compared with the positions of the 18 markers extracted from the acquired images for five levels of fluid. The end point error (EPE), the angular error (AE) and the magnitude error (ME) are averaged across markers and reported for each draining step and each material formulation in Table 2. EPE is the length of the vector difference of the measured displacement vector in the MRI scans and the correspondent one computed in ABAQUS. AE is the angular error calculated on the above-mentioned vectors and provides a method to measure the deviation from the direction of deformation (Barron et al. 1994; Fleet and Jepson 1990). ME is the difference in magnitude between the measured and simulated vectors. Maximum and minimum error values (max/min) are also reported in Table 2, indicating the markers at which these occur (id), along with the standard error of the mean (sem). Figure 7 also shows the displacement vector as a function of CSF (in volume percentage) for a limited number of significant markers. In each sub-figure, the MRI measurements and the model results for the three different material formulations are plotted.

**Table 2:** Average (avg), Standard Error of the Mean (sem) and minimum and maximum values (max/min) of the AE, EPE and ME calculated between MRI scans measurements and model simulations at each draining step (percentage of the volume of CSF lost at each step)

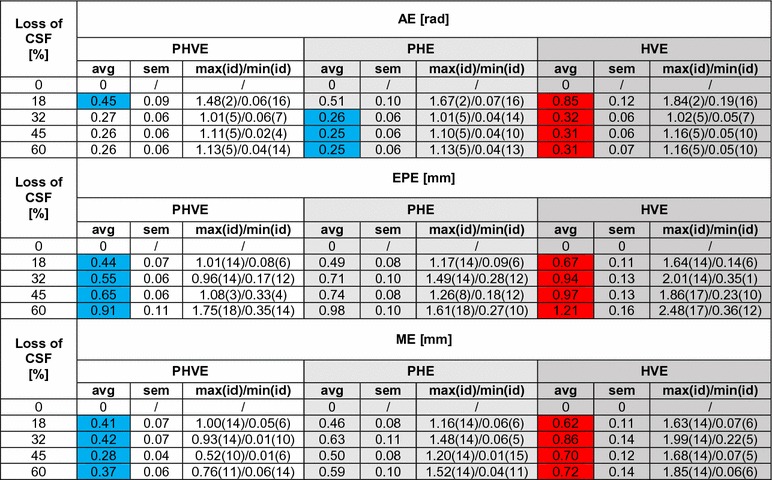	

The markers at which the minimum and maximum error occurs are also reported in parenthesis (id). Please refer to Fig. 2 for the complete markers mapping of the brain phantom. The blue cells indicate the minimum average errors for each draining step across the three models; the red cells indicate the maximum average errors

## Discussion and conclusion

The results obtained comparing three different material formulations proved that a hybrid poro-hyper-viscoelastic description provides the best match with the shift measurements carried out on a synthetic surrogate designed to mimic the mechanical behaviour of the brain tissue. The AE shows the highest value in the first step, remaining, however, below 10%. This occurs for all the material formulations. In fact, during the first steps the majority of the markers were still submerged and thus presented a negligible displacement. Additionally, due to the segmentation process and the MRI resolution, very small mismatches between the model and the real spatial coordinates were present. These discrepancies were considered by the AE as displacement vectors and computed in the averaged value, causing an increase in the overall error. The AE converges to a lower value in the subsequent steps, reaching 0.26 and 0.25 rad for the PHVE and PHE, respectively. The poro-hyper-viscoelastic shows the minimum value for the EPE, while the hyper-viscoelastic shows a deviation of about 1 mm, which represents the resolution of our segmentation. The small mismatch between the model and the measurements can be explained considering two aspects: firstly, for each MRI marker the nearest point of the tetrahedral mesh was considered for the comparison, thus introducing a small error that influenced the EPE; secondly, the AE seems to affect all the material formulations in the same way. This might be due to a small misalignment of the apparatus with respect of the gravity vector. Both these aspects will be further investigated to improve the result of the simulation. The PHVE also exhibits the lowest ME value, confirming that simulating the contribution of both fluid–solid interactions and viscoelastic characteristics in the solid matrix leads to the best approximation of the mechanical behaviour of the hydrogel and therefore of the brain tissue. This is also highlighted in Fig. 7 where the PHVE results exhibit a closer match on all the MRI markers, especially in the areas subjected to higher deformations (frontal lobes, markers 8, 13, 14, 16).

Considering as example the most severe CSF loss condition (60% in Table 2), the AE is largest on the posterior area of the model (marker 5), indicating that there is a small mismatch in the directions along which the phantom and the model are deforming. However, this region is not of interest in any particular surgical scenario, being positioned far away from the craniotomy site. The minimum AE is instead recorded in the upper/frontal part of the brain (especially for PHVE, marker 14), indicating that the three models are accurately predicting the direction of deformation in this areas. Furthermore, frontal lobes represent regions of interest, being in the immediate proximity of the craniotomy site. The EPE is consistently bigger in the right hemisphere (markers 18 and 17) across the three models, while the PHVE model best predicts the deformation near the craniotomy (marker 14), according to the EPE metric. The ME confirms the previous finding, identifying the PHVE with the minimum error in the craniotomy’s proximity, and both the PHE and HVE with the maximum values in the same region. Looking at the average values, the HVE model behaves consistently worse than the two phase models overall (red cells in Table 2).

It is worth noticing that the average ME errors for the PHVE model are significantly different from both the ME errors for the PHE model ($p\,<\,0.05$) and the VHE model ($p\,<\,0.01$). However, the AE and EPE metrics failed to show a significant difference between the averages of the errors. This may be caused by (i) a roughly similar shift direction (AE) across the three models and (ii) a less robust metric definition for the EPE. The significance of the metrics was tested with a standard Student’s two-sample t test. The image segmentation and reconstruction process is limited by the resolution of the MRI scan (1 mm). Therefore, there exists an uncertainty of about 0.5 mm in each spatial direction, which affects the error estimation. This is certainly a limitation of the present study. However, we believe that this does not undermine the overall conclusions of the work, as the PHVE material formulation provides better results consistently (EPE and ME), for each segmented marker. In particular, in the areas affected by large deformations (frontal lobes, markers 8, 13, 14, 16), the PHVE formulation shows a shift prediction which is always well within the segmentation uncertainty ($\pm \,0.5\hbox {mm}$, Fig. 7). Additionally, the PHE and VHE formulation predictions fall outside this confidence range.

The geometrical complexity of the brain requires an automatic mesh generation algorithm to carry out the discretization in a reasonable amount of time. There are many automatic mesh generation algorithms using tetrahedral elements (Owen 2001; Viceconti and Taddei 2003), but not for automatic hexahedral mesh generation. Unfortunately, standard tetrahedral elements exhibit locking when the material is incompressible (Joldes et al. 2009b), and usually, a fine mesh is needed to obtain results of sufficient accuracy (Abaqus software and user manual version 6.13 2013; Borges et al. 2014). Abaqus provides an improved C3D4H tetrahedral formulation (H stands for hybrid) that avoids volumetric locking when incompressibility is required. These elements have already been successfully used in recent biomechanical modelling (Gao et al. 2015). A simple 3D model was designed to investigate the effect of locking in our model when using the C3D4H tetrahedral elements. The brain shape was simplified to a spherical geometry of comparable size (diameter 16 cm). The sphere was fixed for one quarter of its height and tilted of 30 degrees with respect to the vertical direction to reproduce the position of the brain model. The HVE material formulation in Table 1 was used since this was the only formulation involving a fully incompressible solid matrix. Afterwards, a vertical gravity load was applied and the displacement field produced by the spherical model was observed. Using a simple geometry allowed us to automatically mesh it in Abaqus by using both tetrahedral and hexahedral elements.

In the Abaqus hexahedral solid elements, the strain operator provides constant volumetric strain throughout the element. This constant strain prevents mesh locking when the material response is approximately incompressible. Furthermore, the Abaqus manual suggests using reduced integration elements when dealing with nearly incompressible materials (reduced integration on the volumetric terms). Therefore, the C3D8RH elements were used for the hexahedral mesh and compared with the results obtained by the C3D4H tetrahedral mesh. The same seeding length was used for both the meshes (0.16 mm), which was comparable with the average element length in the brain model.

The model meshed with C3D8RH elements took 7664 s to converge, while the one with C3D4H only 502 s.

The models showed a difference of about 0.01 mm in the maximum values of the displacement mapping which can be considered negligible. Therefore, in our particular loading scenario, mesh size and magnitude of deformations, the C3D4H tetrahedral elements effectively prevent locking and save computational time. However, hexahedral elements remain the safest option and caution should be exercised when one is forced to use tetrahedral meshes.

It was shown that the model hereby presented is capable of reproducing the shift phenomenon due to loss of CSF in a phantom apparatus with high accuracy and no intraoperative guidance. Since the phantom reproduces the brain deformations during surgery (Forte et al. 2016a), the model has the potential to reproduce the brain shift in real scenarios and become a valuable tool for surgical training and pre-operative planning.

The material formulation comparison was only possible using a controlled environment (the physical model), which allowed us to rule out many factors that would have introduce uncertainties in the scenario. Therefore, both the fluid and the hyper-viscoelastic solid phase should be considered when modelling nonlinear viscoelastic, porous soft materials. Since the brain tissue is included in this class of materials (Cheng and Bilston 2007; Franceschini et al. 2006), the finding hereby reported should be taken in consideration when modelling brain tissue. Furthermore, being the hydrogel a good surrogate material for the brain tissue at moderate strain rates (Forte et al. 2016a), the values reported in Table 1 can be confidently used as material parameters in indentation and gravity-driven brain shift modelling. Our results also appear to be in line with the work carried out by McGarry et al. (2015) in the context of elastography, where the authors conclude that a two phase constitutive law is more suitable to simulate the mechanical response of the brain tissue at low loading rates. The brain shift phenomenon is indeed known to be characterized by low deformation rates (Nabavi et al. 2000), allowing fluid transport to take place inside the organ.

The poro-hyperelastic and hyper-viscoelastic formulation could be optimized in order to improve their accuracy. For instance, smaller coefficients in the hyperelastic part of the material formulations would lead to a more complaint behaviour which would certainly increase the shift deformation under gravity and therefore have a better match with the measurements. However, the aim of the paper is to demonstrate how, starting from the experimental characterization of the material, a more complex formulation (namely PHVE) can achieve better predictions without further material calibrations. Furthermore, changing the material coefficients in the poro- hyperelastic and the hyper-viscoelastic formulations would defeat the purpose of any comparison across constitutive models. Following the approach we used, we can confidently state that the mismatches between the different models’ results are purely due to their constitutive laws.

The authors would like to point out that the layers of meninges have not been replicated in the current phantom. Consequently, the “free draining” boundary condition at the surface of the phantom material was chosen in our models. Although one may want to improve the phantom in order to obtain a more accurate representation of the real organ, the boundary conditions chosen here are those which are most representative for our experimental set-up. Since the hydrogel is porous and there is no feature put in place to resist draining, water is allowed to flow in and out of the phantom brain.

Future works will include the design of 3D patient-specific brain models with increasing grade of details (inclusion of tentorium, membranes, ventricles, etc.) and evaluate them against intraoperative measurements by means of US and stereo-cameras tracking systems.

Once anatomically improved, the life-sized phantom could also be tested to perform realistic measurements for the validation and advancement of computational modelling of traumatic brain injury by direct comparison with experimental data (Bayly et al. 2012).
